# The Effect of PUFA-Rich Plant Oils and Bioactive Compounds Supplementation in Pig Diet on Color Parameters and Myoglobin Status in Long-Frozen Pork Meat

**DOI:** 10.3390/molecules23051005

**Published:** 2018-04-25

**Authors:** Ewelina Pogorzelska-Nowicka, Jolanta Godziszewska, Jarosław O. Horbańczuk, Atanas G. Atanasov, Agnieszka Wierzbicka

**Affiliations:** 1 Department of Technique and Food Development, Faculty of Human Nutrition and Consumer Sciences, Warsaw University of Life Sciences (WULS-SGGW), Warsaw, Nowoursynowska Street 159 c, 02-776 Warsaw, Poland; jolanta_godziszewska@sggw.pl (J.G.); agnieszka_wierzbicka@sggw.pl (A.W.); 2 Institute of Genetics and Animal Breeding, Polish Academy of Sciences, 05-552 Jastrzębiec, Poland; olav@rocketmail.com (J.O.H.); atanas.atanasov@univie.ac.at (A.G.A.); 3 Department of Pharmacognosy, University of Vienna, Althanstrasse 14, 1090 Vienna, Austria

**Keywords:** PUFA-rich oils, vitamin E, Se, freezing storage, meat color, pork

## Abstract

The study evaluated the effect of pig diet supplementation with rapeseed or linseed oil, and vitamin E or selenium, or both vitamin E and selenium on color parameters and myoglobin content of pork *Semimembranosus* muscle after long-term freezing storage during nine months. The influence of the type of the bioactive compounds added to pig diet on the content of myoglobin or oxymyoglobin, metmyoglobin and deoksymyoglobin in *Semimembranosus* m. was also assessed. The results indicate that the presence of oils rich in polyunsaturated fatty acids (PUFA) in pig diet improves the color of pork meat. Supplementation of dietary plant oils or dietary oils with antioxidants tended to increase significantly the concentration of oxymyoglobin and decrease the concentration of metmyoglobin in meat compared to the control group. The highest content of oxymyoglobin was observed in meat obtained from pigs fed diets with linseed oil. The best color scores (highest a* parameter) was noted for rapeseed oil group (with no addition of antioxidants). In conclusion, the addition of antioxidants to pigs’ forage supplemented with PUFA-rich oils is not recommended in order to improve color of long-term frozen pork.

## 1. Introduction

Meat color is one of the main factors influencing consumers’ purchase decisions [1]. Consumers associate meat color with freshness and wholesomeness. Hence, if the color is not attractive, the meat is rejected during selection at the point-of-sale [2]. The color of meat depends largely on myoglobin content and the ratio of its chemical forms [3]. Myoglobin in meat exists in three forms: oxygenated oxymyoglobin (OxyMb), oxidized metmyoglobin (MetMb) and reduced deoxymyoglobin (DeoMb) [4]. Oxymyoglobin gives the meat bright red-pink color highly preferred by consumers. As a result of oxidative changes, oxymyoglobin is transformed to metmyoglobin of grey-brown color, which is associated by consumers with a meat of poor quality [5]. It is noteworthy that pork contains relatively a lot of saturated fatty acids. The consumption of those fats is associated with increased risk of diet-related diseases [6,7]. The adverse fatty acid profile of meat can be changed by incorporation of oils rich in unsaturated fatty acids (UFA) into animal diet including pigs [8,9,10]. It has been proved that substances formed by the oxidation of fatty acids mediate the process of oxymyoglobin-metmyoglobin oxidation, leading to adverse color change [11]. For this reason, farmers increasingly introduce UFA with antioxidants into pig diet. Different scientific data documented an influence of long-term freezing storage (up to 36 weeks) on physical and chemical attributes of meat including pork [12,13,14,15,16,17,18]. There is also ample information about effects of addition of bioactive compounds and plant oils (linseed oil, rapeseed oil, vitamin E, selenium) on those parameters in meat [8,19,20,21,22,23,24,25]. For example, it was presented that usage of antioxidants such as vitamin E or organic selenium as a component of pigs’ fodder inhibited oxidative changes in meat during storage [19]. Furthermore, Wojtasik-Kalinowska et al. [26] showed that addition of linseed oil, vitamin E and selenium to pigs’ fodder prevented the formation of sulfur compounds in pork *Longissimus dorsi* muscle after long-term freezing. On the other hand, Brodowska et al. [27] indicated that supplementation of pig diet with vitamin E and/or selenium beside the effect on volatile compound profile, did not affect lipid oxidation of *Semimembranosus* muscle (m) after long-term freezing storage. However, there is a shortage of studies on the influence of the application of oils rich in unsaturated fatty acids (UFA) and/or bioactive compounds such as antioxidants (vitamin E, selenium) in pig diet in relation to with long-term freezing storage on the color of pork.

Therefore, the aim of the study was to evaluate of the effect of fodder supplementation with rapeseed oil or linseed oil, and vitamin E or selenium, or both vitamin E and selenium on color parameters and myoglobin content of pork *Semimembranosus* muscle after long-term freezing 9 months storage time. Moreover, in parallel, the ratio of myoglobin forms in the samples was characterized.

## 2. Results

The pH values of analyzed samples ranged from 5.85 to 6.05, which indicated that the pork was of normal quality (no meat with PSE or DFD deficiency was identified). Simultaneously, the basic composition analysis of each sample was performed. Obtained data showed that content of water, protein and fat was in the range of 67–70%, 19–20%, 7–10%, respectively. The results demonstrate that all pork samples had similar quality. Bearing this in mind, we decided to analyze color components and myoglobin content by statistical analyses of data obtained for samples from nine experimental groups, but in three different configurations (Table 1). This approach allowed us to formulate a universal conclusion about the effect of the addition of oils rich in unsaturated fatty acids (UFA) or UFA plus antioxidants to pigs’ fodder on color components and myoglobin content of pork *Semimembranosus* muscle after long-term freezing storage.

### 2.1. Differences in Color Components and Myoglobin Content between Meat Obtained from Pigs Fed Fodder with the Addition of Oils Rich in Unsaturated Fatty Acids (UFA) and Oils Rich in UFA + Antioxidants

In the first statistical approach, the effect of the addition of oils rich in unsaturated fatty acids (UFA) and UFA plus antioxidants to pigs’ fodder on color and myoglobin of pork meat were analyzed. In the Table 2 color parameters of *Semimembranosus* m. from Control, Supplemented group and Control oil group are presented. Pork meat from the Control oil group resulted in a darker (decreased L* value) and more red (increased a* value) color in comparison to the Control and Supplemented groups. Furthermore, meat from the Control oil group was characterized by a significantly (*p* < 0.05) higher chroma (C*) value, which means more vivid color and less close to grey among all the tested groups. Moreover, the current results showed no noticeable differences in hue angle between the groups.

**Figure 1:**
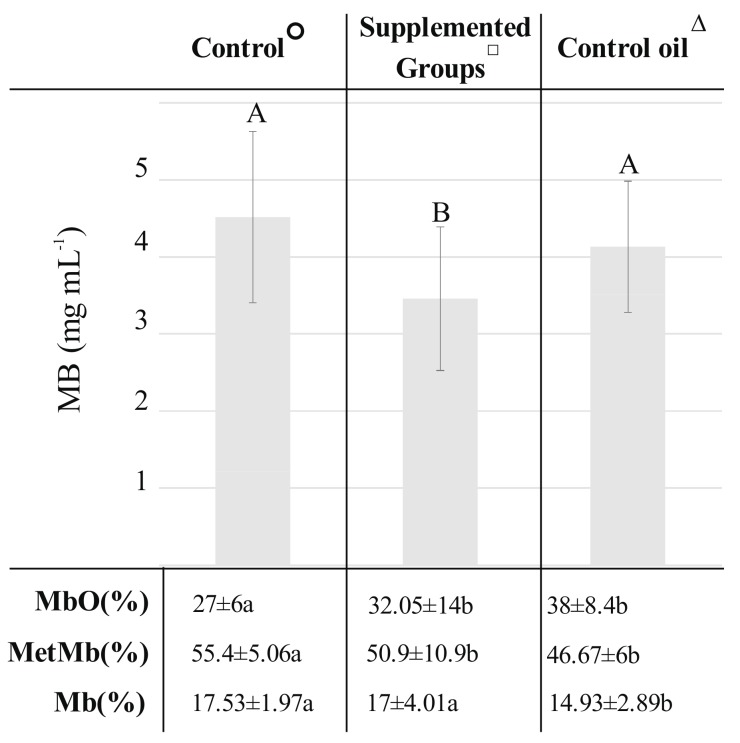
Total myoglobin content and myoglobin chemical forms percentage in meat obtained from different experimental groups: Control°: basal diet with no supplementation; Supplemented Groups^□^: all supplemented groups including: R1: 3% of rapeseed oil (RO), 100 (mg·kg^−1^) of vitamin E (V_E_), 1 mg·kg^−1^ of selenium (Se), R2: 3% of rapeseed oil, 1 (mg·kg^−1^) of Se, R3: 3% of rapeseed oil 100 (mg·kg^−1^) of V_E_, L1: 3% of linseed oil, 100 (mg·kg^−1^) of V_E_, 1 (mg·kg^−1^) of Se, L2: 3% of linseed oil, 1 (mg·kg^−1^) of Se, L3: 3% of linseed oil, 100 (mg·kg^−1^) of V_E_; Control oil^∆^: two control oil groups RO: 3% of rapeseed oil and LO: 3% of linseed oil. Small letters indicate statistical differences between analyzed groups (*p* ≤ 0.05); Capital letters indicates differences between groups in total myoglobin content presented as mg·mL^−1^.

Myoglobin content in analyzed samples according to the Ist statistical approach is presented in Figure 1. There was no observed correlation between a* color parameter and the total myoglobin content in pork subjected to long-term freezing storage. It is noteworthy that, the total myoglobin content was significantly higher in the Control and Control oil groups than in the oils plus antioxidants supplemented groups. Supplementation of dietary oils (Control oil) or dietary oils with antioxidants (Supplemented groups) tends to increase significantly the concentration of oxymyoglobin and decrease concentration of metmyoglobin in meat compared to the Control group (Figure 1).

The obtained data showed that the Control oil group, regarding color components, differ from other analyzed groups. These differences were not observed in myoglobin content. Taking all above into account, in the next step we decided to examine if both oils: rapeseed and linseed have the same effect on color parameters and myoglobin content.

### 2.2. The Impact of the Type of Plant Oil Addition to Fodder of Pigs on Color Parameters and Myoglobin Content of Pork Semimembranosus m.

In the second statistical approach, we compered the effect of rapeseed oil and linseed oil addition to pigs’ fodder on color parameters and myoglobin content in pork meat. After nine months of freezing storage of meat obtained from pigs fed with rapeseed (RO) and linseed oil (LO), we noticed significant differences between those groups in the a* and C* color parameters (Table 3). 

Statistically significant differences between these groups, in the L*, b* as well as h° components, were not observed. Furthermore, we compered obtained results of color components for RO and LO groups, respectively, with color data of Control group (Table 2). Statistically significant differences were detected for parameters: L* for LO group, a* for RO and LO group and C* for RO and LO group (Table 4).

**Figure 2:**
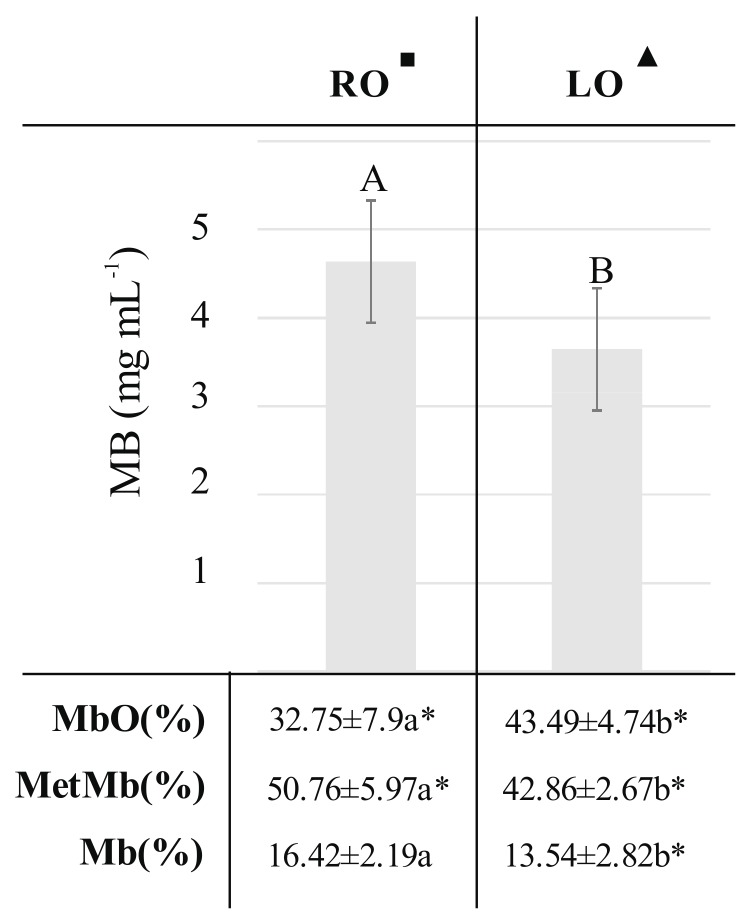
Total myoglobin content and myoglobin chemical forms percentage in meat obtained from different experimental groups: RO^■^: 3% of rapeseed oil; LO^▲^: 3% of linseed oil. Small letters indicate statistical differences between analyzed groups (*p* ≤ 0.05). Capital letters indicates differences between groups in total myoglobin content presented as mg·mL^−1^. Data marked with * sign were additionally compared with Control group (basal diet) and were significantly different.

Even though differences in a* and C* components between RO and LO group were observed, we assumed that there is no effect of oil type on those color parameters. We conclude that based on the comparison of those components between Control oil (both groups RO and LO summed up) and control group where difference was as twice as large. Thus, it can be stated that it is more important whether the oil has been added to the feed than what type of plant oils has been used as an additive. On the other hand, the obtained results revealed that the type of oil used as an additive to pigs fodder is important, when L* color component is consider. Simultaneously, the total myoglobin content and concentrations of myoglobin forms in RO and LO groups were analyzed (Figure 2). 

We found differences in myoglobin content and the ratio of myoglobin forms between those groups. In RO pork we observed a higher total content of myoglobin, while for LO a higher percent of oxymyoglobin. It is also noteworthy that both groups were characterized by a higher oxymyoglobin content in comparison with the Control group. The analyses performed suggested that the type of oil in pig fodder had an impact on the total myoglobin content and relative content of myoglobin forms in pork samples. 

Taking into account, that the total myoglobin content in meat from Supplemented groups was different from the total myoglobin content in meat from Control and Control oil group (Figure 1), we decided to verify whether the addition of specific antioxidants caused differences in color and myoglobin content. 

### 2.3. The Effect of an Addition of Different Antioxidants to Pigs’ Fodder on Color Parameters and Myoglobin Content in Pork Meat 

The incorporation of certain antioxidants into pigs’ diet had a significant impact on the differences in the L*, a*, b* and C* color parameters of meat between the supplemented groups, as shown in Table 4. Meat obtained from pigs fed with oil and selenium (Se) was characterized by the lowest L* color parameter, while a*, b* and C* values decreased in groups fed with oils and vitamin E (V_E_), simultaneously hue angle was not affected by dietary treatment. Despite these results, no differences in color parameters between Control group and V_E_Se, Se, V_E_ groups were observed, except for the b* color parameter, which was lower in the V_E_ group than in Control group.

**Figure 3:**
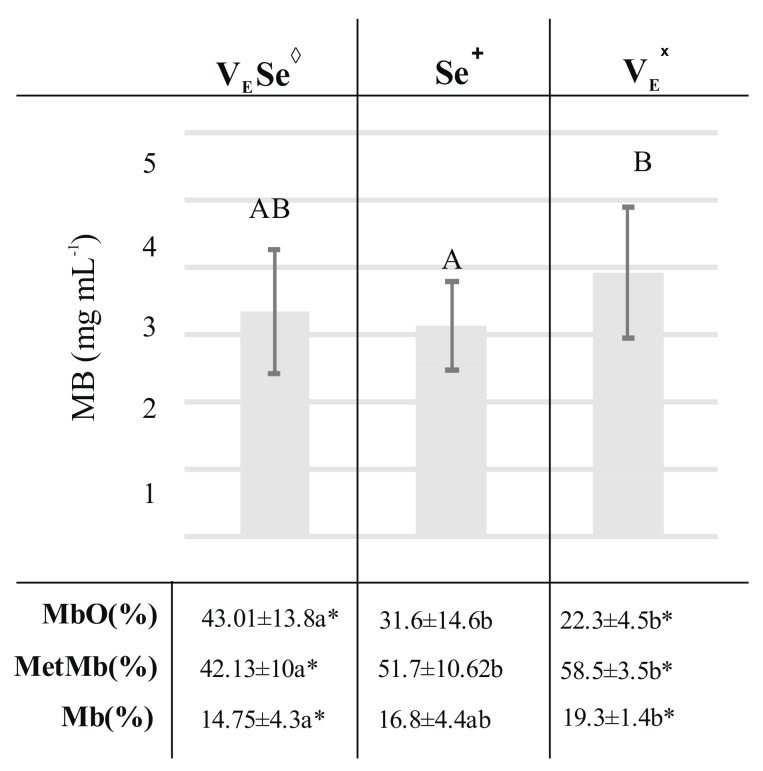
Total myoglobin content and myoglobin chemical forms percentage in meat obtained from different experimental groups: V_E_S_E_^◊^: two supplemented groups: R1: 3% of rapeseed oil, 100 (mg·kg^−1^) of vitamin E (V_E_), 1 (mg·kg^−1^) of selenium (Se) and L1: 3% of linseed oil, 100 (mg·kg^−1^) of V_E_, 1 (mg·kg^−1^) of Se; Se^+^: two supplemented groups: R2: 3% of rapeseed oil, 1 (mg·kg^−1^) of Se and L2: 3% of linseed oil, 1 (mg·kg^−1^) of Se; V_E_^ˣ^: two supplemented groups: R3: 3% of rapeseed oil, 100 (mg·kg^−1^) of V_E_ and L3: 3% of linseed oil, 100 (mg·kg^−1^) of V_E_. Small letters indicate statistical differences between analyzed groups (*p* ≤ 0.05). Capital letters indicates differences between groups in total myoglobin content presented as mg·mL^−1^. Data marked with * sign were additionally compared with Control group (basal diet) and were significantly different.

In this statistical approach, the total myoglobin content, as well as the percentage of myoglobin forms was analyzed. The highest content of oxymyoglobin was observed in meat obtained from pigs fed diets with both vitamin E and selenium (V_E_Se) (Figure 3). 

A particularly significant difference was observed for the V_E_ groups, where the OxyMb content was almost twice as low as in V_E_Se group. At the same time, the highest levels of metmyoglobin and deoxymyoglobin were shown in meat from the Se and V_E_ groups. The differences in the total myoglobin content were also observed for V_E_ and Se groups. Meat from pigs fed fodder with vitamin E had higher level of myoglobin than meat from pigs fed fodder with the addition of Se. Data indicated that the type of antioxidant applied in pigs’ fodder had an impact on color parameters, the total myoglobin content, as well as the percentage of myoglobin forms in pork *Semimembranosus* m.

## 3. Discussion

Literature data indicates that many factors, such as fodder supplementation, storage type, time of storage, packaging method or a type of analyzed muscle have impact on color parameters as well as on the myoglobin content, or concentration of myoglobin forms in pork meat [28,29,30,31,32]. This is noteworthy because meat color is for consumers an important choosing factor. According to the National Pork Producers Council [33] there are five color quality categories of pork meat established based on L* color values: 1st pale greyish (L* around 61), 2nd greyish pink (55), 3rd reddish pink (49), 4th dark reddish pink (43), 5th purplish red (37) and 6th dark purplish red (31). The categories from 3 to 4 were chosen as highly acceptable by consumers. On the basis of the mentioned NPPC’s color categories, the pork *Semimembranosus* m. from the Control oil group after nine months of freezing storage, were characterized as of best color rating (significantly lower L*). Nonetheless at the same time meat from all the examined groups was classified as of desirable color. However, the data obtained by Juarez et al. [34] on the influence of flaxseed oil diet on the color of pork, are inconsistent with our findings. In those studies, it was shown that meat obtained from pigs fed with PUFA-rich oil is lighter, as measured by L* color parameter. 

Simultaneously, the obtained results demonstrated that not only type of applied fodder affected on L* color component of the long frozen meat, but also on a* and C* color parameters. Applied statistical approaches revealed that oils rich in unsaturated fatty acids (UFA) added to pigs’ fodder had a positive effect on a* and C* color parameters of *Semimembranosus* m. Contrary, combined addition of oils rich in unsaturated fatty acids (UFA) with antioxidants to pigs’ fodder did not improve color parameters of pork *Semimembranosus* m. subjected to long-term freezing storage (comparing with meat from Control oil group). This finding is opposite to the observation made by Corino et al. [28]; Haak et al. [29] and Sobotka et al. [32], who found no effect of dietary oils on meat colour and partly in agreement with Okrouhlá [35], who suggested an impact on the b* color parameter. However, they analysed meat samples subjected to different storage conditions, which could change color parameter as Xia et al. [30] noted.

The color parameters in fresh pork are presumed to be linked directly with myoglobin content, as previous studies have documented [36]. Therefore, the total myoglobin content, as well as concentrations of myoglobin forms in each statistical approach were examined. The obtained results showed that used additives in pigs’ fodder had different effect on concentration of myoglobin forms in pork *Semimembranosus* m. after long-term freezing. The total myoglobin content in the meat was also affected by dietary treatment. The data presented that the total myoglobin content was significantly higher in meat from the Control and Control oil group than in the Supplemented group. The differences could result from myoglobin functions in vivo. Garry and Mammen [37] noted that myoglobin serves in vivo as a scavenger of reactive species. In those groups there were no addition of antioxidants and elevated myoglobin content could take over their functions. Thus, the concentration of both myoglobin forms (high oxymyoglobin and low metmyoglobin) in meat from the control oil group is the same like in meat from the Supplemented group. Similar results were obtained by Haak et al. [29] after 3 days of refrigerated storage. In turn, differences in the total myoglobin content and concentration of myoglobin forms in pork *S.* m after long-term freezing storage between RO and LO groups could be a consequence of other factors influencing the antioxidative status of added oils such as the content of tocopherol, polyphenols or plant origin sterols [38,39]. Results of the total myoglobin content and concentrations of its forms in meat from V_E_Se, Se, V_E_ groups were performed due to the inconsistent effect of vitamin E supplementation on oxymyoglobin oxidation noted in some previous studies. Tang et al. [40] advocate that α-tocopherol reduces MetMb formation. In turn, Lee et al. [41] did not observe an impact of vitamin E on OxyMb oxidation in pork, but in beef the effect was observed [42]. The differences may result from the primary sequence of pork and beef myoglobin. Bovine myoglobin contains thirteen histidine residues, while pork has only nine. Those nucleophilic residues have the ability to bind with secondary products of lipid peroxidation, which makes myoglobin more susceptible to oxidation and, in turn, it may affect different vitamin E capability for preventing OxyMb oxidation in pork and beef meat [43]. Correlations between color parameters (L*, a*, b*, C*, h°) and the total content of myoglobin as well as OxyMb, MetMb, Mb forms were performed in order to verify if the parameters of color are associated with concentration of myoglobin forms and/or the total content of myoglobin in long-frozen pork. Spearman rank correlation (*p* < 0.05) revealed only a weak positive correlation between OxyMb and the L* color parameter (R = 0.19) as well as a weak negative correlation between MetMb and L* (R = −0.18) and between Mb and L* (R = −0.2). The obtained results confirmed that in pigs’ *S*. m. after long-term freezing storage the a*, b*, C* and h° color parameters are not directly associated with the total content of myoglobin or with the concentration of specific myoglobin forms. Contrary data was showed by Lindahl et al. [44]. The researchers indicated that for fresh pork loin L*, a*, C* as well as h° color parameters are influenced by myoglobin forms. The lack of correlations in our study might be connected with the long-term freezing storage of the muscles, which contributes to structural damage of meat tissue due to myosin denaturation, osmotic removal of water, mechanical damage, and cross-linking and aggregation of myofibrillar proteins [45]. Therefore, an important challenge for the future will be to analyze correlations between indicators of structural damage of pork muscle after long-term freezing storage and the total content of myoglobin, concentrations of OxyMb, MetMb, Mb forms as well as color components.

## 4. Conclusions

The applied statistical approaches allowed us to cluster treatment groups in terms of similar effects on the color parameters, myoglobin content and the concentration of its forms. Analysis showed that the meat obtained from groups supplemented with PUFA-rich oils, subjected to long-term freezing storage had most attractive color (lower L* and higher a* color parameter) compared to both control group and groups supplemented with oils and antioxidants. The analysis of the color in pork meat revealed the influence of the type plant oils on this parameter. Regarding the content of myoglobin or OxyMb, MetMb, Mb forms in *Semimembranosus* m, the type of the compounds added to pig diet is also important. Additionally, no correlations between the content of myoglobin or its forms and color parameters were documented, what could have resulted from the structural changes of meat formed by long-term storage under cold conditions. 

## 5. Materials and Methods

### 5.1. Experimental Design and Sample Collection

The material for analysis consisted of *Semimembranosus* m. taken from crossbreeds of Polish Landrace and Duroc pigs with the initial body weight of 60 ± 5 kg. A total number of animals included in the experiment was 108. The animals were randomly selected and divided into nine treatment groups (12 animals in each group). For the further analysis from each group six animals were randomly selected (54 animals). The pigs in the control group were fed a basal diet, whereas the rest of the groups (eight groups) were fed a basal diet supplemented with additional compounds as follows: RO (3% of rapeseed oil), LO (3% of linseed oil), R1 (3% of rapeseed oil, 100 mg·kg^−1^ of vitamin E (V_E_) and 1 mg of organic selenium (Se)), R2 (3% of rapeseed oil, 1 mg·kg^−1^ of Se), R3 (3% of rapeseed oil, 100 mg·kg^−1^ of V_E_), L1 (3% of linseed oil, 1 mg·kg^−1^ of Se, 100 mg·kg^−1^ of V_E_), L2 (3% of linseed oil, 1 mg·kg^−1^ of Se), L3 (3% of linseed oil and 100 mg·kg^−1^ of V_E_). The basal diet was completely balanced and formulated based on barley grits (360 g·kg^−1^), wheat middling (360 g·kg^−1^), corn grits (100 g·kg^−1^), extracted soybean meal (80 g·kg^−1^), extracted rapeseed meal (40 g·kg^−1^) and vitamin-mineral premix (25 g·kg^−1^). Animals were kept individually in pens with unlimited access to feed and water (each pen equipped with an automatic feeder and nipple drinker), under the same temperature, air speed and humidity conditions (of values respectively: 18–20 °C, 60–70%, 0.2–4 m·s^−1^). After reaching a 100 ± 5 kg of live weight, the pigs were slaughtered at a local provincially-inspected slaughter facility using slaughter technology covering electrical stunning, bleeding in a perpendicular-hanging position and cooling by the single-degree method. After 48 h *post mortem* meat cuts were transported to the laboratory under refrigerated conditions. At the laboratory, four samples (of about 150 g each) were cut aseptically from each muscle of each pig. Two samples were analyzed directly in order to determine the pH values and basic composition (water, fat and protein content). Remaining two slices were vacuum sealed with film of permeability for: 0_2_ TR of 40 cm^3^/m^2^/24 h/bar, CO_2_ TR of 150 cm^3^/m^2^/24 h/bar, water vapour TR of 2.6 g/m^2^/24 h using Cryovac VS26 (Sealed Air Cryovac, Charlotte, NC, USA). Right after packaging samples were frozen at −20 °C and then stored for 9 months. After the storage, the color and myoglobin content, as well as ratio of myoglobin forms was analyzed.

### 5.2. pH Measurement

Measurements of pH values were taken by a portable pH meter Testo 205 (Mera, Warsaw, Poland). The calibration of the pH meter was conducted at the ambient temperature using two buffers solutions (pH = 4.01 and pH = 7.00). The measurements were performed in triplicate for each sample by the direct insertion of glass probe in meat to a depth of about 20 mm.

### 5.3. NIR Measurement

The basic composition of the meat samples was evaluated by the NIR Flex N-500 spectrometer of close infrared radiation (Büchi Labortechnik AG, Flawil, Switzerland) equipped with NIR Ware 1.1, NIR Cal 5.1 spectral analysis software. Before analysis samples (150 g of meat) were evenly homogenized and then were placed in glass Petri dishes and scanned. For each sample, three replicates were taken and averaged. 

### 5.4. Color Measurement 

The meat color was presented in (CIE) L*a*b* system. Measurements were performed on freshly thawed meat after 30 min exposure to atmospheric oxygen in the refrigerator conditions (4 °C). The analysis was conducted on the 30 mm thick slices of *Semimembranosus* muscles using a Konica Minolta Chroma Meter CR-400 (Minolta Co., Ltd., Osaka, Japan) with the head of 8 mm diameter measurement area, previously calibrated with D 65 standard illuminant and 2° standard colorimetric observer. For each sample, results were presented as average values of ten measurements collected on randomly selected areas across the slice surface. Chroma/saturation (C) and hue angle (h°) was calculated according to the equations: C = √[(a*)^2^ + (b*)^2^] and h° = tan^−1^ (b*/a*), where a* and b* were the color readings. The data of color parameters of fresh meat (before freezing) was also measured and presented in Appendix A (Table A1) in order to present initial values.

### 5.5. Preparation of MetMb, DeoMb and OxyMb

Metmyoglobin stock solution was prepared using a pure commercial equine standard (Sigma-Aldrich Ltd., Poznań, Poland). The horse heart myoglobin was dissolved in 40 mM potassium phosphate buffer (pH 6.8) to a concentration of 10 mg·mL^−1^ and oxidized by the addition of potassium ferricyanide (Chempur, Piekary Śląskie, Poland) in a concentration of 5 mg mL^−1^. Afterwards, the oxidation reagent was removed by exhaustive dialysis against 40 mM potassium phosphate buffer. After dialysis, the stock solution was diluted to a concentration of 2.0 mg·mL^−1^. A part of such prepared metmyoglobin stock solution (2 mg·mL^−1^) was reduced to deoxymyoglobin by the addition of potassium dithionite (Avantor Performance Materials S.A., Gliwice, Poland) in a concentration of 0.5 mg·mL^−1^ and flushing with pure nitrogen through the stock solution. Oxymyoglobin form was obtained by flushing pure oxygen through the deoxymyoglobin stock solution. Finally, wavelengths at which maximum absorption occurred for each of myoglobin redox form were measured using Shimadzu UV-1800 spectrophotometer (Kyoto, Japan).

### 5.6. Myoglobin Measurement

Myoglobin extraction was performed according to the 2012 Meat Color Measurement Guidelines (AMSA, Champaign, IL, USA) with some modifications [46]. Five grams of *Semimembranosus* muscle were homogenized with 25 mL of 40 mM potassium phosphate buffer (pH 6.8) for 40–50 s, at 5000 rpm using Wiggenhauser WT 500 homogenizer (Berlin, Germany). The homogenate was placed in an ice bath for 1 h. After cooling, samples were centrifuged at 10,000 rpm (MPW 325R, MPW Med., Warsaw, Poland) for 25 min at 10 °C and filtered through Whatman No.1 filter paper (PHPU Eurochem BGD, Tarnów, Poland). For further purification, the supernatant was clarified through a syringe filter (0.4 µm). The absorbance for each sample was measured at 525 nm (the isobestic point for all myoglobin forms) using Shimadzu UV-1800 spectrophotometer (Kyoto, Japan). To calculate the ratio of the different forms of myoglobin in the sample, the following wavelengths were selected: 504 nm (for MetMb), 557 nm (DeoMb) and 582 nm (OxyMb) maximal absorption values. The relative amounts of chemical forms were calculated using the Tang et al. [47] equations:

[DeoMb] = −0.543R1 + 1.594R2 + 0.552R3 − 1.329

[OxyMb] = 0.722R1 − 1.432R2 − 1.659R3 + 2.599

[MetMb] = −0.159R1 − 0.085R2 + 1.262R3 − 0.520

where R1 = A582/A525, R2 = A557/A525, R3 = A504/A525.

### 5.7. Statistical Analysis

Data were analyzed using procedures of STATISTICA 12 (StatSoft, Tulsa, OK, USA). To verify the normality of data distribution, the Shapiro-Wilk test was conducted. Differences between the treatment groups were verified by Kruskal-Wallis ANOVA followed by multiple comparisons of mean ranks for all groups or Mann-Whitney U test with the *p* ≤ 0.05.

## Figures and Tables

**Table 1:** Configuration of experimental groups in statistical groups in three statistical approaches.

Statistical Approaches		I			II		III		
	Statistical Groups	Control	Supplemented Groups	Control Oil	RO	LO	V_E_	Se	V_E_+
Experimental Groups									
C (basal diet—BS)		x							
RO (BS + rapeseed oil)				x	x				
R1 (BS + rapeseed oil + V_E_)			x		x		x		
R2 (BS + rapeseed oil + Se)			x		x			x	
R3 (BS + rapeseed oil + V_E_ + Se)			x		x				x
LO (BS + linseed oil)				x		x			
L1 (BS + linseed oil + V_E_)			x			x	x		
L2 (BS + linseed oil + Se)			x			x		x	
L3 (BS + linseed oil + V_E_ + Se)			x			x			x

C: basal diet with no supplementation, RO: 3% of rapeseed oil, R1: 3% of rapeseed oil (RO), 100 mg·kg^−1^ of vitamin E (V_E_), 1 (mg·kg^−1^) of selenium (Se), R2: 3% of rapeseed oil, 1 (mg·kg^−1^) Se, R3: 3% of rapeseed oil 100 (mg·kg^−1^) of V_E_, LO: 3% of linseed oil L1: 3% of linseed oil, 100 (mg·kg^−1^) of V_E_, 1 (mg·kg^−1^) of Se, L2: 3% of linseed oil, 1 (mg·kg^−1^) of Se, L3: 3% of linseed oil, 100 (mg·kg^−1^) of V_E_.

**Table 2:** The comparison of the effect of specific groups of compounds on color parameters of *Semimebranosus* m. measured after long-term freezing storage.

Trait	Control° (*n* = 6, N = 10)		Supplemented Groups^□^ (*n* = 36, N = 10)		Control Oil^∆^ (*n* = 12, N = 10)	
	Mean ± SD	Median (Min–Max)	Mean ± SD	Median (Min-Max)	Mean ± SD	Median (Min–Max)
L*	45.55 ± 4.52a	45.33 (36.82–56.44)	44.95 ± 4.77a	44.50 (35.15–60.94)	43.34 ± 3.56b	43.62 (34.35–54.83)
a*	11.87 ± 3.44a	11.59 (6.95–19.59)	11.93 ± 4.30a	11.33 (3.02–26.52)	14.50 ± 3.5b	14.29 (5.61–22.46)
b*	4.53 ± 1.70ab	4.4 (1.49–8.92)	4.21 ± 1.87a	3.84 (0.35–12.53)	5.08 ± 1.99b	4.75 (1.4–11.47)
C*	12.82 ± 3.41a	12.44 (7.49–21.48)	12.73 ± 4.42a	12.11 (4.04–29.33)	15.47 ± 3.61b	15.27 (6.54–25.21)
h°	0.37 ± 0.14	0.35 (0.15–0.77)	0.35 ± 0.13	0.32 (0.07–1.04)	0.34 ± 0.12	0.33 (0.12–0.67)

Notes: Control°—basal diet with no supplementation; Supplemented groups^□^: all supplemented groups including: R1: 3% of rapeseed oil (RO), 100 (mg·kg^−1^) of vitamin E (V_E_), 1 (mg·kg^−1^) of selenium (Se), R2: 3% of rapeseed oil, 1 (mg·kg^−1^) of Se, R3: 3% of rapeseed oil 100 (mg·kg^−1^) of V_E_, L1: 3% of linseed oil, 100 (mg·kg^−1^) of V_E_, 1 (mg·kg^−1^) of Se, L2: 3% of linseed oil, 1 (mg·kg^−1^ ) of Se, L3: 3% of linseed oil, 100 (mg·kg^−1^) of V_E_; Control oil^∆^: two control oil groups RO: 3% of rapeseed oil and LO: 3% of linseed oil. *n*: number of animals, N: number of repetitions; Small letters indicate statistical differences between analyzed groups (*p* ≤ 0.05)

**Table 3:** The comparison of the effect of oil type (linseed or rapeseed) used as additive to pig’ fodder on color parameters in *Semimebranosus* m. after long-term frozen storage.

Trait	RO^■^ (*n* = 6, N = 10)		LO^▲^ (*n* = 6, N = 10)	
	Mean ± SD	Median (Min–Max)	Mean ± SD	Median (Min–Max)
L*	43.92 ± 2.95	44.01 (36.57–54,83)	42,75 ± 4,02 *	42.82 (34.35–51.78)
a*	15.24 ± 2.86a *	15.09 (9.9–22.5)	13.77 ± .93b *	13.8 (5.61–21.3)
b*	5.32 ± 1.84	4.75 (2.43–11.5)	4.85 ± 2.11	4.71 (1.4–9.23)
C*	16.21 ± 3.02a *	16.27 (11.29–25.22)	14.73 ± 4.0b *	14.81 (6.54–23.20)
h°	0.34 ± 0.09	0.32 (0.18–0.6)	0.35 ± 0.14	0.34 (0.12–0.67)

Notes: RO^■^: 3% of rapeseed oil; LO^▲^: 3% of linseed oil; *n*: number of animals, N: number of repetitions; Small letters indicate statistical differences between analyzed groups (*p* ≤ 0.05); Data marked with * sign were additionally compared with Control group (basal diet) and were significantly different.

**Table 4:** Comparisons of color parameters of *Semimebranosus* m. obtained from pigs fed fodder with PUFA-rich oils and V_E_ + Se or Se or V_E_ measured after long-term freezing storage.

Trait	V_E_Se^◊^ (*n* = 12, N = 10)		Se^+^ (*n* = 12, N = 10)		V_E_^ˣ^ (*n* = 12, N = 10)	
	Mean ± SD	Median (Min–Max)	Mean ± SD	Median (min-max)	Mean ± SD	Median (Min–Max)
L*	46.66 ± 4.4a	45.32 (36.66–60.94)	43.83 ± 4.98b	43.38 (35.15–58.62)	45 ± 4.74a	44.75 (35.60–57.26)
a*	11.95 ± 4.22ab	11.41 (3.02–24.91)	11.83 ± 4.22a	12.56 (5.61–26.52)	11.01 ± 4.31b	10.57 (3.91–24.89)
b*	4.29 ± 1.81a	3.88 (1.14–9.73)	4.72 ± 2.06a	4.30 (1.75–12.53)	3.61 ± 1.57b^*^	3.38 (0.33–8.66)
C*	12.8 ± 4.28ab	12.32 (5.5–25.68)	13.78 ± 4.36a	13.29 (5.88–29.33)	11.66 ± 4.41b	11.19 (4.04–26.35)
h°	0.36 ± 0.14	0.33 (0.103–1.035)	0.36 ± 0.13	0.33 (0.159–0.69)	0.33 ± 0.12	0.32 (0.07–0.68)

Notes: V_E_S_E_^◊^: two supplemented groups: R1: 3% of rapeseed oil, 100 (mg·kg^−1^) of vitamin E (V_E_), 1 mg·kg^−1^ of selenium (Se) and L1: 3% of linseed oil, 100 (mg·kg^−1^) of V_E_, 1 (mg·kg^−1^) of Se; Se^+^: two supplemented groups: R2: 3% of rapeseed oil, 1 mg·kg^−1^ of Se and L2: 3% of linseed oil, 1 mg·kg^−1^ of Se; V_E_^ˣ^: two supplemented groups: R3: 3% of rapeseed oil, 100 (mg·kg^−1^) of V_E_ and L3: 3% of linseed oil, 100 (mg·kg^−1^) of V_E_; *n*: number of animals, N: number of repetitions; Small letters indicate statistical differences between analyzed groups (*p* ≤ 0.05); Data marked with ^*^ sign were additionally compared with Control group (basal diet) and were significantly different.

## Appendix A

**Table A1:** The comparison of the effect of bioactive compounds used as additives to pigs’ fodder on color parameters in *Semimebranosus* m. in fresh meat.

Trait	Control° (*n* = 6, N = 10)		Control Oil^∆^ (*n* = 12, N = 10)		V_E_^ˣ^ (*n* = 12,N = 10)		Se^+^ (*n* = 12, N = 10)		V_E_Se^◊^ (*n* = 12, N = 10)	
	Mean ± SD	Median (Min–Max)	Mean ± SD	Median (Min–Max)	Mean ± SD	Median (Min–Max)	Mean ± SD	Median (Min–Max)	Mean ± SD	Median (Min–Max)
L*	45.55 ± 4.52ac	45.33 (36.82–56.44)	43.34 ± 3.56b	43.62 (34.35–54.83)	45.36 ± 4.74a	44.75 (35.6–57.26)	43.83 ± 4.98ac	43.38 (35.15–58.62)	45.66 ± 4.74bc	45.32 (36.66–60.94
a*	11.87 ± 3.44ac	11.6 (6.95–19.55)	14.5 ± 3.5b	14.3 (5.61–22.46)	11.01 ± 4.31ac	10.58 (3.91–24.89)	12.83 ± 4.22a	12.57 (5.61–26.52)	11.95 ± 4.22c	11.41 (3.02–24.91)
b*	4.53 ± 1.7ac	4.4 (0.49–8.92)	5.08 ± 1.99a	4.75 (1.4–11.47)	3.61 ± 1.5c	3.38 (0.33–8.66)	4.72 ± 2.06b	4.31 (1.75–12.53)	4.29 ± 1.81ac	3.88 (1.14–9.73)
C*	12.82 ± 3.41ac	12.45 (7.5–21.49)	15.47 ± 3.61	15.27 (6.54–25.22)	11.61 ± 4.41	11.14 (4.05–26.35)	13.78 ± 4.36	13.33 (5.88–29.33)	12.8 ± 4.28	12.32 (5.5–25.68)
h°	0.57 ± 0.14	0.36 (0.15–0.77)	0.34 ± 0.12	0.33 (0.12–0.67)	0.33 ± 0.12	0.32 (0.07–0.68)	0.36 ± 0.13	0.34 (0.16–0.69)	0.36 ± 0.14	0.33 (0.1–1.04)

Notes: Control°—Basal diet; Control oil^∆^: two groups: RO: 3% of rapeseed oil and LO: 3% of linseed oil; V_E_S_E_^◊^: two supplemented groups: R1: 3% of rapeseed oil, 100 mg·kg^−1^ of vitamin E (V_E_), 1 mg·kg^−1^ of selenium (Se) and L1: 3% of linseed oil, 100 mg·kg^−1^ of V_E_, 1 mg·kg^−1^ of Se; Se^+^: two supplemented groups: R2: 3% of rapeseed oil, 1 mg·kg^−1^ of Se and L2: 3% of linseed oil, 1 mg·kg^−1^ of Se; V_E_^ˣ^: two supplemented groups: R3: 3% of rapeseed oil, 100 mg·kg^−1^ of V_E_ and L3: 3% of linseed oil, 100 mg·kg^−1^ of V_E_. *n*: number of animals, N: number of repetitions. Small letters indicate statistical differences between analyzed groups (*p* ≤ 0.05).
